# Risk factors for internet addiction among Croatian university students during the COVID-19 pandemic: Maja Miskulin

**DOI:** 10.1093/eurpub/ckac130.184

**Published:** 2022-10-25

**Authors:** M Miskulin, N Pavlovic, I Miskulin, J Kovacevic, D Laslo, I Vukoja

**Affiliations:** Department of Public Health, Faculty of Medicine Osijek, University of Osijek, Osijek, Croatia

## Abstract

**Background:**

Following the increased internet use due to the COVID-19 pandemic there have been concerns regarding an elevated risk of developing internet addiction (IA). University students are especially prone to develop IA and risk factors for its development in this population during pandemics are not fully investigated nor understood. This study aimed to identify possible risk factors of IA in the studied population during the ongoing pandemic and to compare it with risk factors in pre-pandemic time.

**Methods:**

In April 2016 and April 2022 a validated, anonymous questionnaire that contained questions regarding demographic data, as well as Young's Internet Addiction Test, was self-administered to a cross-faculty representative student sample of the University of Osijek, Croatia.

**Results:**

The study included 1602 university students median age of 21 years (interquartile range 20-22), 34.5% males, and 65.5% females. There was no statistically significant difference in the median age between the two student samples (p = 0.234). The main reason for internet use (social networking and entertainment and online gaming) was considered the significant risk factor for IA in a studied population in pre-pandemic time (the year 2016) and pandemic time (the year 2022) (p < 0.001). In pre-pandemic time the IA was more frequent in males (p = 0.046) while the difference in IA prevalence between sexes did not exist during pandemics (p = 0.160). During pandemics, the students who did not work during their study had higher proportions of IA (p = 0.021) while there was no difference in IA prevalence among students regarding their working status during the study in pre-pandemic time (p = 0.251).

**Conclusions:**

During the COVID-19 pandemic working status of students has been recognized as the new risk factor for IA in the Croatian university students population. Further studies are needed to identify other possible risk factors for IA in the studied population during pandemics.

**Key messages:**

- The COVID-19 pandemic has changed the set of risk factors for IA among Croatian university students.
- Better understanding of risk factors for IA among university students during the COVID-19 pandemic will enable the development of successful preventive programs for this behavioral addiction.

